# Dynamics of Inorganic Nutrients in Intertidal Sediments: Porewater, Exchangeable, and Intracellular Pools

**DOI:** 10.3389/fmicb.2016.00761

**Published:** 2016-05-26

**Authors:** Emilio Garcia-Robledo, Julio Bohorquez, Alfonso Corzo, Juan L. Jimenez-Arias, Sokratis Papaspyrou

**Affiliations:** ^1^Microbiology Section, Department of Biosciences, Aarhus UniversityAarhus, Denmark; ^2^Departamento de Biología, Facultad de Ciencias del Mar y Ambientales, Universidad de CádizPuerto Real, Spain; ^3^Laboratorio de Microbiología, Departamento de Biomedicina, Biotecnología y Salud Pública, Facultad de Ciencias, Universidad de CádizPuerto Real, Spain

**Keywords:** microphytobenthos, intracellular nutrients, exchangeable nutrients, porewater nutrients, ammonium, nitrate, phosphate, intertidal sediments

## Abstract

The study of inorganic nutrients dynamics in shallow sediments usually focuses on two main pools: porewater (PW) nutrients and exchangeable (EX) ammonium and phosphate. Recently, it has been found that microphytobenthos (MPB) and other microorganisms can accumulate large amounts of nutrients intracellularly (IC), highlighting the biogeochemical importance of this nutrient pool. Storing nutrients could support the growth of autotrophs when nutrients are not available, and could also provide alternative electron acceptors for dissimilatory processes such as nitrate reduction. Here, we studied the magnitude and relative importance of these three nutrient pools (PW, IC, and EX) and their relation to chlorophylls (used as a proxy for MPB abundance) and organic matter (OM) contents in an intertidal mudflat of Cadiz Bay (Spain). MPB was localized in the first 4 mm of the sediment and showed a clear seasonal pattern; highest chlorophylls content was found during autumn and lowest during spring-summer. The temporal and spatial distribution of nutrients pools and MPB were largely correlated. Ammonium was higher in the IC and EX fractions, representing on average 59 and 37% of the total ammonium pool, respectively. Similarly, phosphate in the IC and EX fractions accounted on average for 40 and 31% of the total phosphate pool, respectively. Nitrate in the PW was low, suggesting low nitrification activity and rapid consumption. Nitrate accumulated in the IC pool during periods of moderate MPB abundance, being up to 66% of the total nitrate pool, whereas it decreased when chlorophyll concentration peaked likely due to a high nitrogen demand. EX-Nitrate accounted for the largest fraction of total sediment nitrate, 66% on average. The distribution of EX-Nitrate was significantly correlated with chlorophyll and OM, which probably indicates a relation of this pool to an increased availability of sites for ionic adsorption. This EX-Nitrate pool could represent an alternative nitrate source with significant concentrations available to the microbial community, deeper in the sediment below the oxic layer.

## Introduction

Sediment inorganic nutrients are a key biogeochemical component of aquatic ecosystems. Studying the seasonal and spatial changes in nutrient fluxes and water column concentrations in aquatic systems allow us to explain the ecosystem's productivity. However, interpretation of nutrient data is often difficult due to the complex interactions and overall dynamics of the various sediment nutrient pools. Mineralization of organic matter in the upper layers of the sediment, particularly in shallow environments, releases inorganic nutrients to the pore water (PW) which are exchanged with the water column by diffusive and advective transport processes. Alternatively, nutrients in the pore water can be adsorbed to different degrees to either organic compounds or clay particles in the sediment (Mackin and Aller, 1984; Coelho et al., 2004). The adsorbed fraction is typically referred to as the exchangeable nutrient fraction (EX). In addition, nutrients in the pore water are consumed by sediment microalgae and prokaryotes for assimilatory or dissimilatory purposes.

During the last decade, the capacity of some components of the microbial benthic community to store large concentrations of nutrients intracellularly (intracellular fraction, IC) has been emphasized (Kamp et al., 2015). In a previous study, we established a clear relation between the freeze-lysable inorganic nutrient pool (FLIN) and microphytobenthos (MPB) abundance, which suggested that benthic microalgae are able to accumulate nitrate, ammonium, and phosphate intracellularly (García-Robledo et al., 2010). Kamp et al. (2011), using axenic cultures, demonstrated that benthic diatoms can accumulate nitrate intracellularly, being able to use it to survive during dark and anoxic periods. During an annual study, Stief et al. (2013) showed that benthic diatoms in intertidal sediments of the Wadden Sea also accumulate nitrate. More recently, Yamaguchi et al. (2015) demonstrated that microalgae from intertidal sediments of Ariake Bay (Japan) accumulate both organic and inorganic phosphorus intracellularly.

The PW, EX, and IC nutrient pools are subject most likely to different biogeochemical and ecological controls. Therefore, fractionation of inorganic nutrients in the various sediment pools needs to be addressed both conceptually and methodologically. This will allow identifying the relevant nutrient fractions that should be quantified in aquatic sediments to answer specific biogeochemical problems. The seasonal variations of environmental variables and nutrient supplies in Cadiz Bay are expected to determine the variations of MPB inhabiting the intertidal areas. In a previous study based on only two samplings (summer/winter), García-Robledo et al. (2010) suggested that the magnitude and fractionation of the different nutrients in the PW and IC fractions is likely related to the MPB abundance and activity. For Wadden Sea sediments, Stief et al. (2013) also suggested a link between nitrate pools and MPB variations over a 12 month period; however, no information was provided about other relevant inorganic nutrients such as ammonium or phosphate. Thus, the relative importance of these nutrient pools and their seasonal dynamics remain largely unknown. In addition, the other major nutrient pool in marine sediment, the exchangeable fraction, has not been studied simultaneously with the PW and IC pools before.

The aim of this study was to understand the ecological and biogeochemical importance of each of the three nutrient pools, PW, EX, and IC. We hypothesize that these pools must be functionally linked to the microbial community, particularly to MPB, and therefore will exhibit spatial and temporal variability related to that of biological and abiotic variables in the water column and the sediment. To test this, here we analyze the relationships between chlorophyll content, used as a proxy for MPB abundance, and inorganic nutrients (${\text{NH}}_{4}^{+}$, ${\text{NO}}_{3}^{-}$, ${\text{PO}}_{4}^{3-}$, and ${\text{SIO}}_{4}^{4-}$) changes in the three sediment pools. These relationships were evaluated using data obtained from an intertidal sediment area of Cadiz Bay (Spain) with depth (upper 1 cm) during a period of 18 months. Although, intertidal sediments are exposed to periodical immersion and emersion periods which have been shown to affect the inorganic nutrient dynamics (Rocha and Cabral, 1998; Rocha, 1999; Hou et al., 2005), our study was not focused on the short-term effects produced by the tidal cycle but rather on seasonal changes. Thus, samplings were carried out always during emersion periods in order to simplify the sampling strategy. In addition, in the present study, we focused on the pools that we considered the most readily available for the microorganisms or in direct physicochemical equilibrium with the pore water. For the first time the PW, IC, and EX nutrient pools were extracted using a sequential procedure, allowing us to compare directly the different ammonium, nitrate, phosphate nutrient pools and to determine their relationship with the seasonal MPB cycle. In addition, as a first approach to understand the ecological and biogeochemical importance of each of these nutrient pools, we explore the relationships of their spatial and seasonal variability with biological and abiotic variables in the water column and the sediment.

## Materials and methods

### Sampling site and collection of samples

Sediment samples were collected monthly at an unvegetated intertidal site in Trocadero Island (36° 31′ 09″ N, 6° 12′ 01″W; inner Cadiz Bay, Spain) from January 2008 to June 2009. Sediment was silty mud and no macroalgae or seagrass detritus were observed at the surface of the sampled sediment. A detailed description of the sampling point can be found in Papaspyrou et al. (2013). From December 2008 to February 2009, when green macroalgae (tubular *Ulva* sp.) covered large patches of the sediment surface, care was taken to collect sediment samples in non-covered areas. Sediment cores were collected using cut-off syringes (i.d. 1.6 cm, *n* = 7) from three random sediment plots (1 × 1 m^2^) separated 10–15 m between them (21 cores in total). Sediment collection was always performed during low tide shortly after the sediment got exposed. We selected cores of small diameter to facilitate subsequent sediment slicing at millimeter scale. Another two sediment cores with a larger diameter (plexiglas core liners, i.d. 5.4 cm) were collected in each one of the three random plots for microsensor measurements (6 cores in total). Water column samples (20 mL, *n* = 3) were collected from the adjacent subtidal area. All cores and water samples were maintained on ice and transported immediately to the laboratory. Water samples were filtered immediately after arriving to the laboratory and stored frozen (−20°C) until later analysis. Nutrient concentrations (${\text{NH}}_{4}^{+}$, ${\text{NO}}_{2}^{-}$, ${\text{NO}}_{3}^{-}$, ${\text{PO}}_{4}^{3-}$, and ${\text{SiO}}_{4}^{4-}$) were measured as described below.

### Microsensor measurements

After arriving to the laboratory, the large sediment cores were maintained in an aquarium illuminated with halogen lamps at 800 μE m^−2^ s^−1^ (average annual PPF value) under a 12 h light:12 h dark photoperiod. The aquarium was filled with seawater collected *in situ*. In order to measure steady state profiles, sediment cores were maintained overnight in the aquarium at room temperature (20–22°C) and microsensor measurements were performed the following day. Oxygen profiles (*n* = 3–4 per core) were measured in light and in darkness in each of the six sediment cores using O_2_ microelectrodes (25 μm tip size, UNISENSE A/S, Denmark). Light conditions were maintained for at least 2 h prior to measuring the profiles. Potential net primary production (pPn) and dark respiration (pRd) were calculated from the O_2_ gradient at the diffusive boundary layer of the light and dark profiles, respectively (Revsbech and Jorgensen, 1986; Kühl et al., 1996). Potential Net Daily Production (NDP), expressed as daily net rates (mmol O_2_ m^−2^ d^−1^), was calculated as the sum of the product of pPn and pRd rates multiplied by the corresponding number of hours in day or night (natural photoperiod), respectively, each month.

### Sequential nutrient pools extraction

Once in the laboratory, the small diameter sediment cores (i.d. 1.6 cm) were sliced using a razor blade and a caliper into 1 millimeter sections down to 10 mm depth by slowly pushing up the syringe piston. This fine sectioning was possible due to the consolidated nature of the sediment found at our sampling point and the small diameter of the cores. Slices of four cores from the same depth and plot were pooled to obtain a sufficiently large amount of sample and homogenized.

Nutrient pools were then extracted sequentially from the sediment using the following sequence:

- Pore water (PW) extraction: pore water was extracted from the sediment by centrifugation at 8600 × g for 20 min at 4°C. All the supernatant was extracted recording the volume obtained (0.15–0.6 mL) and preserved at −20°C.
- Intracellular (IC) or Freeze-Lysable Inorganic Nutrient (FLIN) extraction: in order to break the MPB cells and release their intracellular content, sediment was freeze-thawed three times. Freezing was achieved at −80°C and thawing at room temperature. Freeze-thawed sediment was then resuspended in 4 mL of artificial sea water (40 PSU), centrifuged at 8600 × g for 20 min at 4°C, and the supernatant collected and preserved at −20°C.
- KCl-Exchangeable fraction (EX): Immediately after the extraction of IC nutrients, 2 mL of a 2M KCl solution were added to the samples. Sediment was resuspended and incubated at 4°C for 30 min (Holmboe and Kristensen, 2002) and the supernatant extracted and preserved at −20°C.

Nutrient samples were diluted initially to a total volume of 4 mL with artificial sea water (for PW and IC samples) or Milli-Q water (for EX samples). Concentrations of ${\text{NH}}_{4}^{+}$, ${\text{NO}}_{2}^{-}$, ${\text{NO}}_{3}^{-}$, ${\text{PO}}_{4}^{3-}$, and ${\text{SiO}}_{4}^{4-}$ were measured on a Technicon Autoanalyzer (TRAACS-800) following standard protocols (Grasshoff et al., 1983). Further dilutions were done when concentrations exceeded the calibration curve range.

### Photosynthetic pigments and organic matter measurements

Immediately after EX extraction, pigments were extracted from the same sediment samples. Chlorophylls (Chl) were extracted in 100% methanol buffered with MgCO_3_ overnight at 4°C in darkness (Thompson et al., 1999). Extracts were centrifuged at 2200 × g for 10 min and absorbance was measured using a spectrophotometer (Unicam UV/Vis UV2^®;^). Chl *a* and *c* were estimated after Ritchie (2008). It is likely that the nutrients extraction procedure may have degraded partly the chlorophylls in the samples and chlorophyll values here may underestimate *in situ* values. Sediment samples were then dried at 60°C for 24 h to determine the dry weight (g dw) and porosity in order to normalize the measured variables.

The remaining sediment cores (*n* = 3, per station) were sliced in 2 mm sections. Samples were dried at 60°C for 24 h and organic matter content (OM) was estimated as loss on ignition (Heiri et al., 2001).

### Calculations of nutrient pools content

Nutrient concentrations (μmol L^−1^, equivalent to nmol mL^−1^) measured in the extracts were converted to nutrient content in the different pools (nmol cm^−3^). The contribution of the residual water remaining in the sediment after each supernatant extraction step was also taken into account in the calculations of the nutrient pools using the following equations:

(1) $$\begin{array}{r} PW\;\left(nmol\;cm^{-3}\right)=\left[\frac{C_{PW}\times \;V_{T}}{W}\right]\times \rho_{D} \end{array}$$

(2) $$\begin{array}{r} IC\;\left(nmol\;cm^{-3}\right)=\\ \;\left[\frac{C_{IC}\times \left(V_{ASW}+V_{RPW}\right)-\left(C_{PW}\times V_{RPW}\right)}{W}\right]\times \rho_{D} \end{array}$$

(3) $$\begin{array}{r} EX\;\left(nmol\;cm^{-3}\right)=\\ \left[\frac{C_{EX}\times \left(V_{KCl}+V_{RIC}\right)-\left(C_{IC}\times V_{RIC}\right)}{W}\right]\times \rho_{D} \end{array}$$

where, *C*_*PW*_ is the nutrient concentration in the porewater (nmol mL^−1^); *C*_*IC*_ is the nutrient concentration in the IC extraction (nmol mL^−1^); *C*_*EX*_ is the nutrient concentration in the KCl extraction (nmol mL^−1^); *V*_*T*_ is the total water volume of the sediment sample (fresh–dry weight) (mL); *V*_*ASW*_ and *V*_*KCl*_ are the volume of artificial sea water added for the IC and KCl extractions (4 and 2 mL, respectively); *V*_*RPW*_ and *V*_*RIC*_ are the residual water volume remaining after the extractions of pore water and IC (mL); *W* is the dry weight of the sediment sample (*g dw*); ρ_*D*_ is the dry density of the fresh sediment (0.66 g dw cm^−3^).

Considering standard limits of detection of 0.1 μM for ${\text{PO}}_{4}^{3-}$ and 0.5 μM for ${\text{NH}}_{4}^{+}$ and ${\text{NO}}_{3}^{-}$ for the analysis of the diluted PW and the IC and EX extracts and an average sample weight of 0.5 g dw, the detection limits for the pools was 0.5 nmol cm^−3^ for ${\text{PO}}_{4}^{3-}$ and 2.6 nmol cm^−3^ for ${\text{NH}}_{4}^{+}$ and ${\text{NO}}_{3}^{-}$.

### Statistical analysis

The relationship between the different sediment fractions of ${\text{PO}}_{4}^{3-}$, ${\text{NH}}_{4}^{+}$, and ${\text{NO}}_{3}^{-}$ with their corresponding water column concentrations, MPB biomass, OM content, NDP, and climatological variables (i.e., air temperature, rainfall, and irradiance) were initially analyzed by Spearman correlation analysis to produce two correlation matrices. Monthly averages (average of the 3 profiles per month) of each nutrient pool, photosynthetic pigments, and OM profiles (*n* = 17 months × 10 depths = 170) were used to calculate correlations between the variables including both spatial (vertical) and temporal variability. In order to establish correlations with other environmental (climatological and water column nutrients) and biological (NDP and Chl *a* and *c*) variables, the upper 4 mm of the OM and nutrients pools were integrated. Variability was then reduced to temporal changes and thus, Spearman correlations (*n* = 17 months × 3 plots = 51) could be calculated between the nutrient pools and the rest of the variables.

## Results

### Porewater and water column nutrients

**Figure 1:**
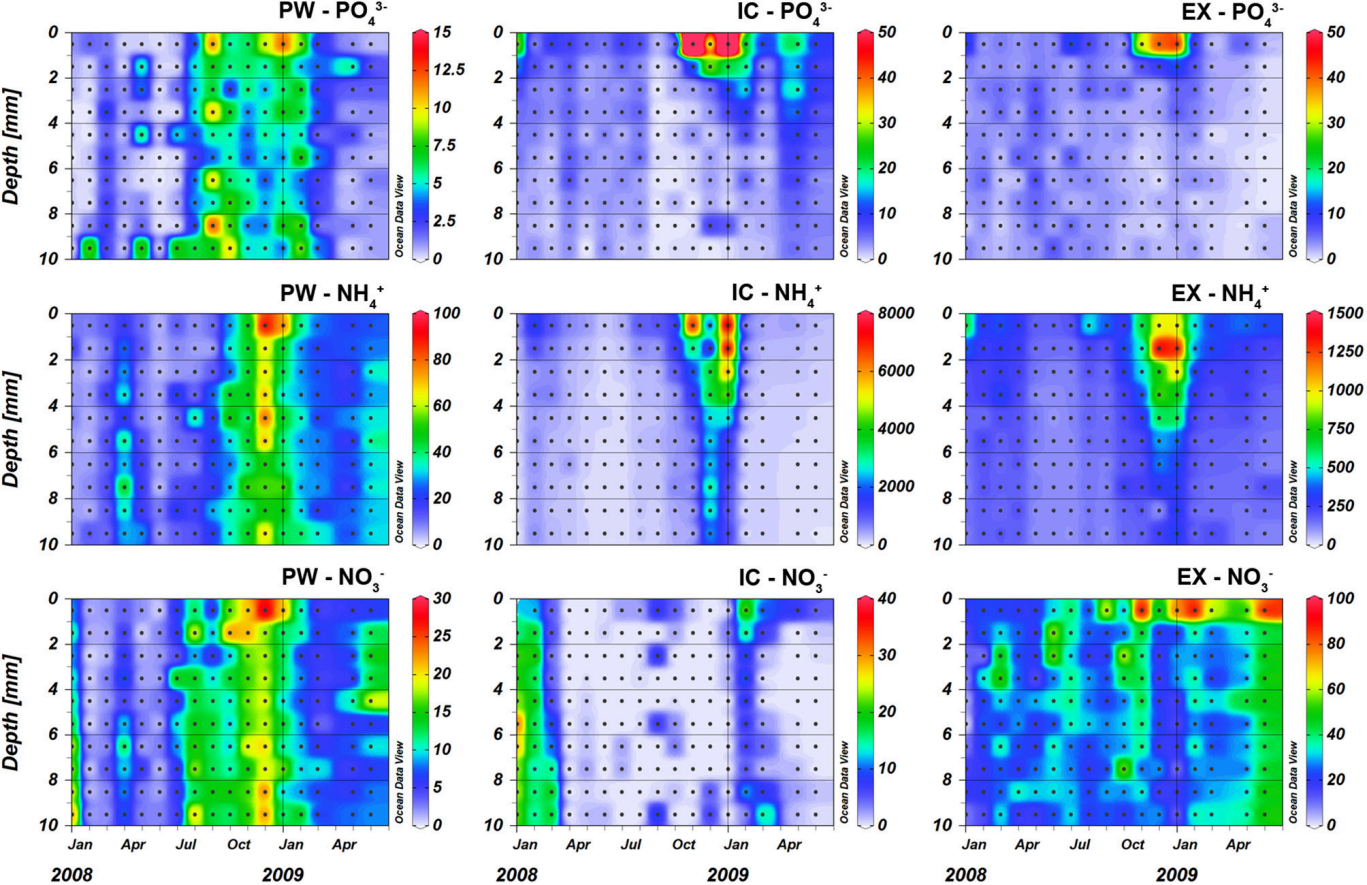
**Nutrient content (nmol cm^**−3**^) in the porewater (PW), intracellular (IC), and KCl exchangeable (EX) fractions at the sampling station on Trocadero Island, Bay of Cádiz from January 2008 to June 2009**. Notice the different scales of each graph. Data are mean of 3 sediment plots.

**Figure 2:**
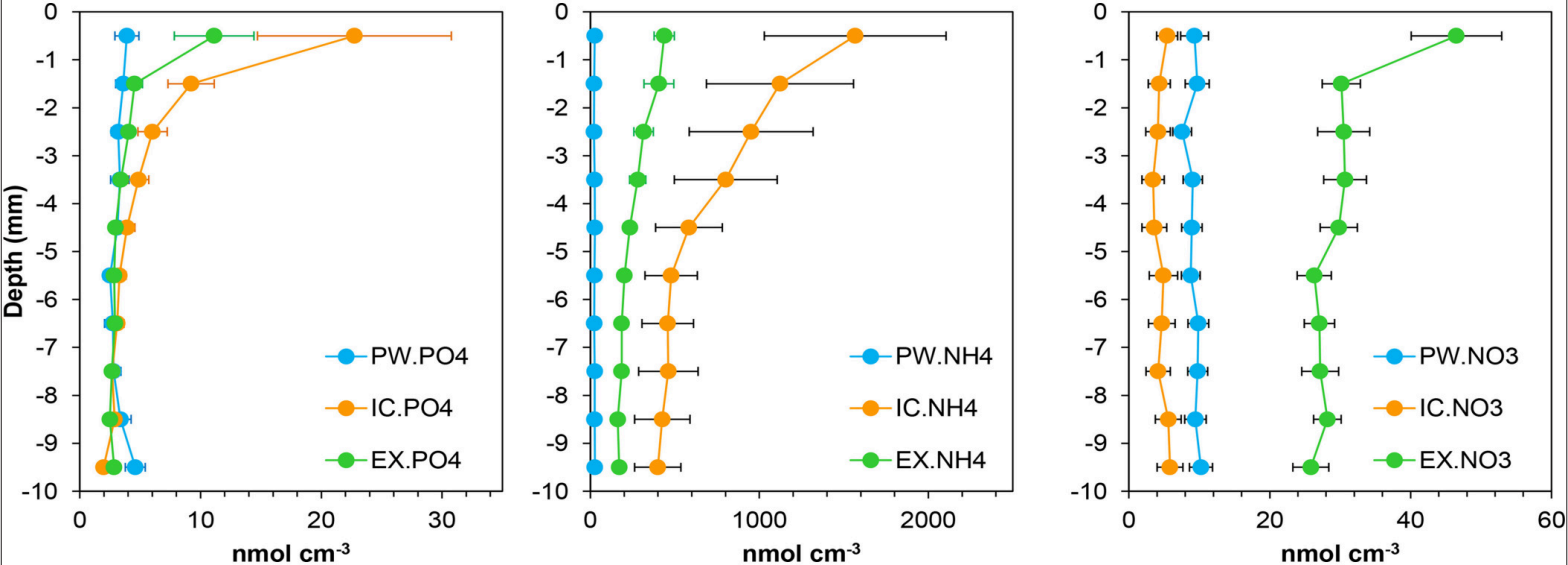
**Mean profiles of the nutrient pools content in the porewater (PW), intracellular (IC), and KCl exchangeable (EX) fractions at the sampling station on Trocadero Island, Bay of Cádiz**. Data are mean of 17 months ± SE (from January 2008 to June 2009).

**Figure 3:**
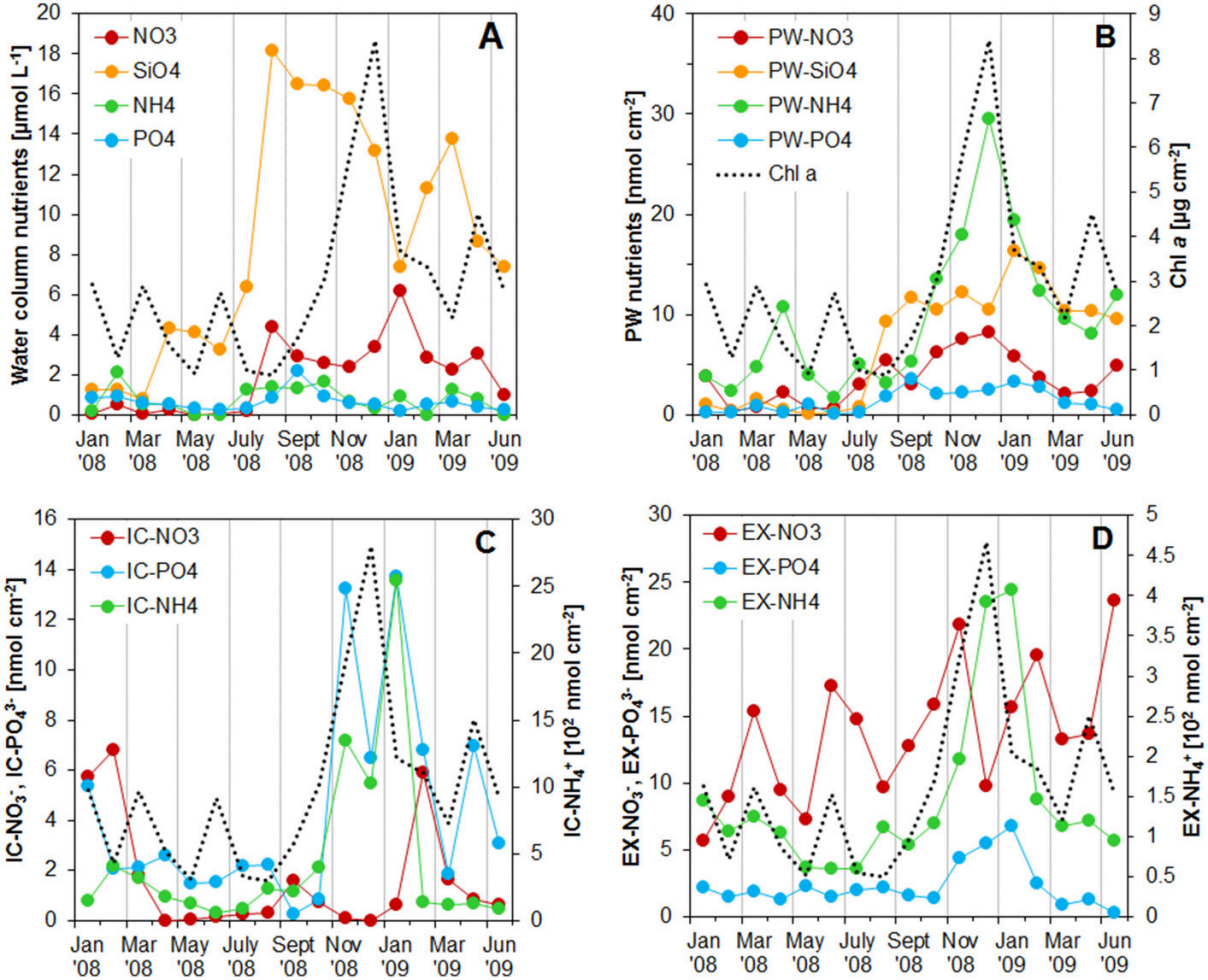
**(A)** Concentration of nutrients in the water column at the sampling station on Trocadero Island, Bay of Cádiz, from January 2008 to June 2009. **(B–D)** Integrated nutrient pools in the first 4 mm of the sediment: **(B)** porewater, **(C)** intracellular, and **(D)** exchangeable pools. Integrated Chlorophyll a content was also drawn in all the graphs for comparison. Notice the different scale (10^2^ nmol cm^−3^) for intracellular and exchangeable ammonium pools in **C** and **D**.

Porewater nutrient (PW) contents showed pronounced spatial and seasonal changes for all the analyzed nutrients (Figures 1, 2). In general, nutrient contents were low and increased during autumn, peaking at the sediment surface in December 2008 with values of 89.4, 9.2, and 28.4 nmol cm^−3^ for PW ammonium (${\text{PW-NH}}_{4}^{+}$), phosphate (${\text{PW-PO}}_{4}^{3-}$), and nitrate (${\text{PW-NO}}_{3}^{-}$), respectively. Nutrients content integrated in the first 4 mm showed the same pattern, with high values during autumn and very low values during spring-summer months (Figure 3B). The content of ${\text{PW-SiO}}_{4}^{4-}$ in the first 4 mm of the sediment increased after July 2008, from relatively low values of 1 nmol cm^−3^ to values higher than 10 nmol cm^−3^, peaking in January 2009 (Figure 3B).

Nutrients in the water column varied throughout the sampled period (Figure 3A). Silicate was the most abundant nutrient with a maximum concentration of 18.0 μM and a mean concentration of 8.8 μM. Nitrate was the second most abundant nutrient with maximum concentration of 6.2 μM during January 2009. Nitrate concentrations were almost zero during the first half of 2008 and increased after August 2008 to values around 3.0 μM, decreasing again in June 2009. Phosphate and ammonium concentrations were generally low, remaining below 1 μM. Higher ammonium concentrations were measured during February 2008 (2.1 μM) and from July to October 2008 (1.3–1.7 μM). Phosphate also increased during the same periods, reaching values of 0.9 μM in February 2008 and 2.2 μM in November 2008.

**Figure 4:**
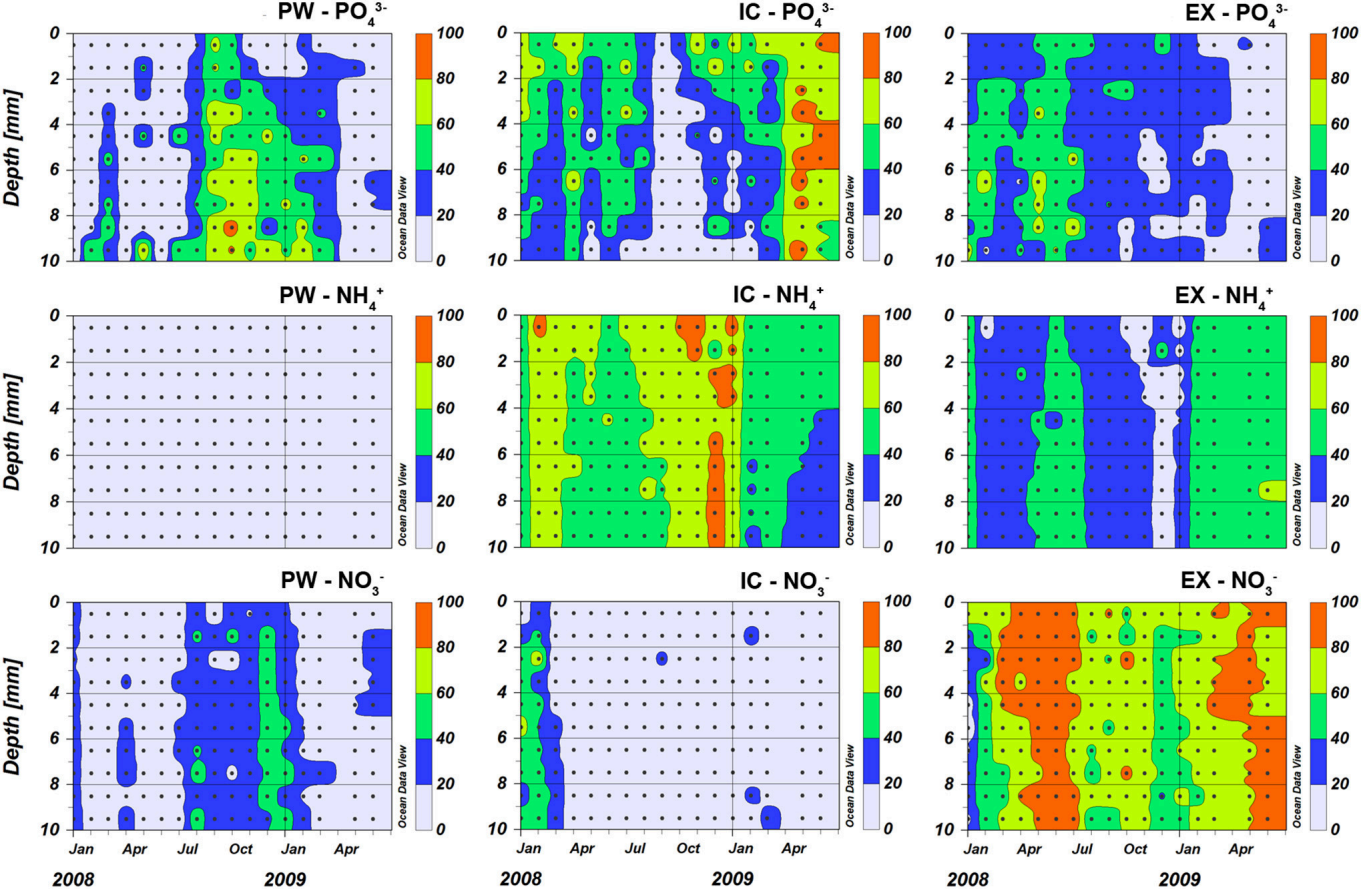
**Nutrient pools content in the porewater (PW), intracellular (IC) and KCl exchangeable (EX) fractions expressed as percentage (%) of the total nutrient pool at the sampling station on Trocadero Island, Bay of Cádiz, from January 2008 to June 2009**. In order to facilitate comparison the data were represented using a discrete color gradient with steps of 20%.

The contribution of ${\text{PW-PO}}_{4}^{3-}$, ${\text{PW-NH}}_{4}^{+}$, and ${\text{PW-NO}}_{3}^{-}$ to the total corresponding nutrient content in the sediment varied considerably, both with depth within the sediment and along the seasonal cycle (Figure 4). ${\text{PW-NH}}_{4}^{+}$ accounted for only 4.1 ± 3.1% (mean ± SD) of the total ammonium content, being around 3% throughout the studied period and increasing to mean values of 8% in spring-summer 2009. ${\text{PW-PO}}_{4}^{3-}$ comprised on average 29 ± 24% of the total. It increased generally with sediment depth and was more important during autumn and winter (September 2008 to February 2009) when it was above 60%. ${\text{PW-NO}}_{3}^{-}$ contribution to sediment nitrate was similar to ${\text{PW-PO}}_{4}^{3-}$; its average contribution was 21 ± 14% (mean ± SD) being highest during winter, when it accounted for up to 50% of the total sediment nitrate.

### Intracellular nutrients

The Intracellular (IC) pool was highly variable both spatially and temporally. The three main nutrients analyzed (${\text{IC-PO}}_{4}^{3-}$, ${\text{IC-NH}}_{4}^{+}$, and ${\text{IC-NO}}_{3}^{-}$) showed a common profile; IC contents peaked at the sediment surface, decreasing exponentially with depth to relatively constant values below 4–5 mm depth (Figures 1, 2). ${\text{IC-NH}}_{4}^{+}$ and ${\text{IC-PO}}_{4}^{3-}$ showed the highest concentrations at the sediment surface from November 2008 to January 2009 (6934-7672 and 117.4–97.5 nmol cm^−3^ for ${\text{IC-NH}}_{4}^{+}$ and ${\text{IC-PO}}_{4}^{3-}$, respectively). It is noteworthy that both nutrients showed two peak values, one just before (November 2008) and one after (January 2009) the Chl *a* maximum (Figures 1, 3). In contrast, ${\text{IC-NO}}_{3}^{-}$ content was minimal during the Chl *a* peak, and was highest before and after Chl *a* content started to increase (i.e., in February 2008, September 2008, and February 2009). During these months, the 0–4 mm integrated ${\text{IC-NO}}_{3}^{-}$ content varied from 1.6 to 6.8 nmol cm^−2^, whereas it remained at values below 1 nmol cm^−2^ during the rest of the year (Figure 3).

${\text{IC-NH}}_{4}^{+}$ and ${\text{IC-PO}}_{4}^{3-}$ were the main sediment nutrient pool, contributing on average 59 ± 15% and 40 ± 24%, respectively (Figure 4). These pools were dominant close to the sediment surface, with their contribution decreasing progressively with depth. The ${\text{IC-NH}}_{4}^{+}$ pool showed a clear seasonal pattern, with higher contribution during autumn-winter months and lower in spring-summer. In contrast, little nitrate was found in the IC fraction in the sediments of Cádiz Bay (9 ± 15%). ${\text{IC-NO}}_{3}^{-}$ was relatively important only in the 2008 winter season, with contributions higher than 40% on occasions.

### Exchangeable nutrients

Exchangeable (EX) fractions of ammonium (${\text{EX-NH}}_{4}^{+}$) and phosphate (${\text{EX-PO}}_{4}^{3-}$) showed, to some extent, a similar pattern to that of the IC ones. Highest values were observed within the upper two millimeters of the sediment (1324–1365 nmol cm^−3^ for ${\text{EX-NH}}_{4}^{+}$ and 40.8–43.3 nmol cm^−3^ for ${\text{EX-PO}}_{4}^{3-}$) in December 2008 and January 2009 (Figure 1), whereas values were low below 2–3 mm depth for ${\text{EX-PO}}_{4}^{3-}$ and 5–6 mm for ${\text{EX-NH}}_{4}^{+}$. During the rest of the year, values were relatively low with no clear vertical structure. The exchangeable nitrate fraction (EX-NO${\text{EX-NO}}_{3}^{-}$) also showed maximum values at the sediment surface (46.5 ± 6.4 nmol cm^−3^) and lower values at deeper depths (Figures 1, 2). Seasonal variations of ${\text{EX-NO}}_{3}^{-}$ were less pronounced when compared to the other nutrients; highest contents were measured in November 2008, February 2009, and June 2009.

${\text{EX-PO}}_{4}^{3-}$ represented a similar percentage of total ${\text{PO}}_{4}^{3-}$ as ${\text{IC-PO}}_{4}^{3-}$, making up 31 ± 15% of the total ${\text{PO}}_{4}^{3-}$ pool (Figure 4). The ${\text{EX-NH}}_{4}^{+}$ fraction was always higher that ${\text{PW-NH}}_{4}^{+}$ at all depths, but equal or lower than ${\text{IC-NH}}_{4}^{+}$, comprising 37 ± 13% of the total. ${\text{EX-NH}}_{4}^{+}$ fraction showed its lowest contribution in autumn. Surprisingly, ${\text{EX-NO}}_{3}^{-}$ was the most abundant nitrate fraction in the sediment (69 ± 17%), with its relative contribution being highest from March to June in both years.

### Photosynthetic pigments, organic matter, and net production

**Figure 5:**
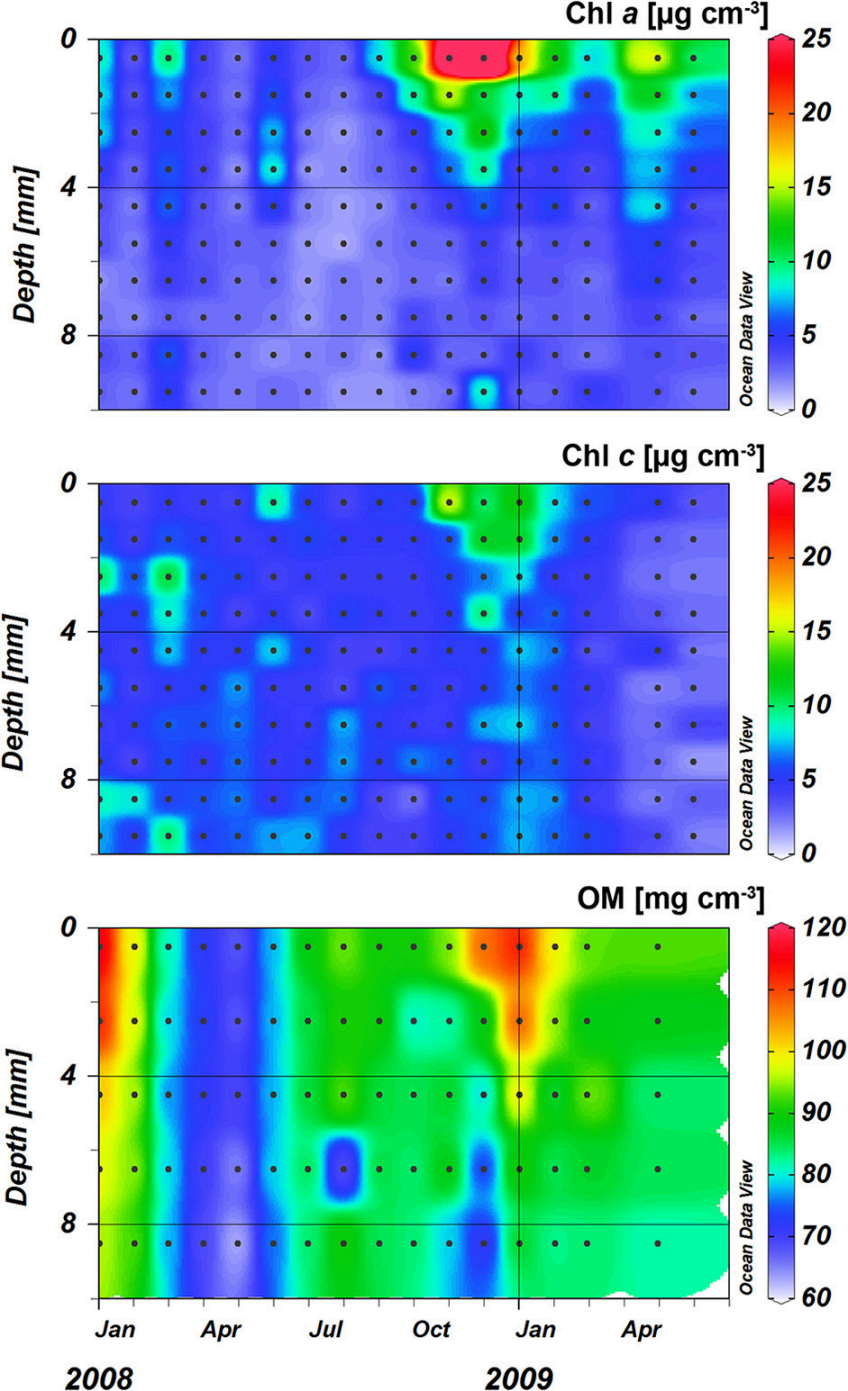
**Chlorophyll ***a*** (Chl ***a***), chlorophyll ***c*** (Chl ***c***), and organic matter (OM) profiles at the sampling station on Trocadero Island, Bay of Cádiz, from January 2008 to June 2009**. Data are mean of 3 sediment plots.

**Figure 6:**
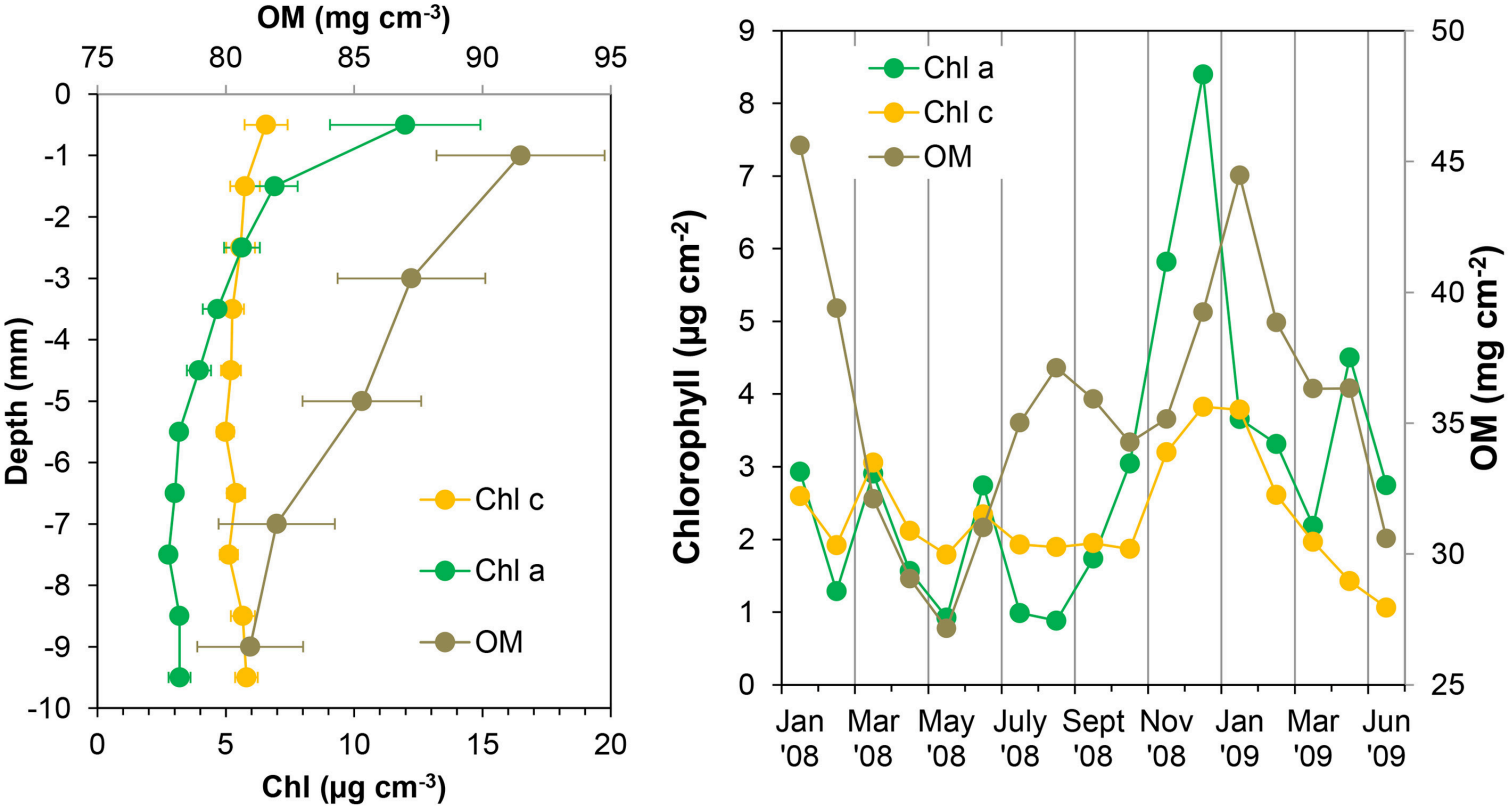
**Chlorophyll ***a*** (Chl ***a***), chlorophyll ***c*** (Chl ***c***), and organic matter (OM) mean profiles (left panel) and integrated values of the first 4 mm of the sediment (right panel) at the sampling station on Trocadero Island, Bay of Cádiz, from January 2008 to June 2009**. Data are mean ± SD.

Chlorophyll (Chl) content varied significantly over time and with depth in the sediment, especially in the upper few millimeters of the sediment (Figure 5). During the late autumn and winter months, chlorophylls content was highest at the sediment surface (12.0 ± 2.9 and 6.6 ± 0.8 μg cm^−3^ for Chl *a* and Chl *c*, respectively); chlorophylls decreased exponentially with depth to relatively low concentrations remaining constant below 4–5 mm (3.2 ± 0.4 and 5.1 ± 0.4 μg cm^−3^ at deeper layers for Chl *a* and Chl *c*, respectively; Figure 6).

Concentrations at the sediment surface (< 4 mm) showed a clear seasonal pattern (Figures 5, 6). Maximum concentration for both Chl *a* and *c* was found during late autumn (November-December 2008), whereas minimum values were measured during spring and summer (April-August 2008 and May-June 2009). A secondary Chl *a* maximum was detected in March 2009 without, however, a parallel increase in Chl *c* (Figure 6).

Organic matter (OM) content showed similar variations over time and with depth to those of photosynthetic pigments (Figures 5, 6). Highest OM content was found in the first few millimeters of sediment during the end of autumn-beginning of winter (November to February; Figure 5). Maximum integrated values were observed in January 2008 and 2009 (45.6 and 44.5 mg cm^−2^, respectively; Figure 6), whereas minimum values during in June 2008 and 2009 (31 and 30.6 mg cm^−2^, respectively). On average, OM content at the surface was 91.5 ± 3.3 mg g^−1^, decreasing progressively to 80 ± 2.1 mg g^−1^ at 10 mm depth (Figure 6). However, the temporal pattern of OM presented certain discrepancies compared to that of photosynthetic pigments; in January 2009 and for the period from July to August 2008, peaks in OM were not reflected in similar peaks in the photosynthetic pigments.

**Figure 7:**
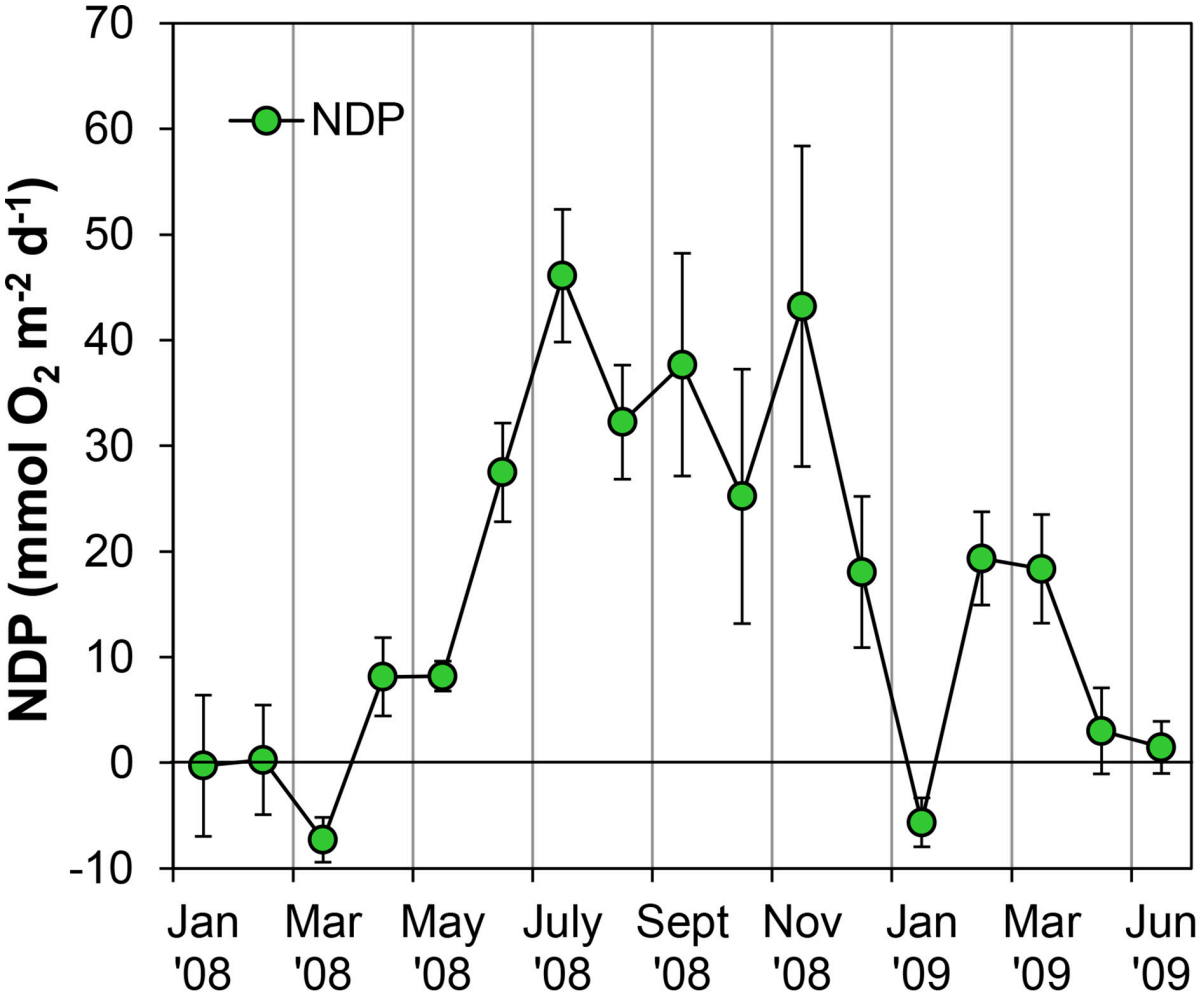
**Evolution of potential Net Daily Production (NDP) metabolism measured as Oxygen flux at the sediment-water interface using oxygen microsensors at the sampling station on Trocadero Island, Bay of Cádiz, from January 2008 to June 2009**. Data are mean of 4–6 sediment cores ± SD.

Sediment cores were always net autotrophic when illuminated at 800 μE m^−2^ s^−1^ in the lab, resulting in positive NDP rates during most of the sampling period (Figure 7). During late winter and spring, NDP rates were relatively low; lower than 10 mmol O_2_ m^−2^ d^−1^ and even negative on occasions. NDP increased during summer and autumn months, reaching maximum rates of 43–46 mmol O_2_ m^−2^ d^−1^ in July and November 2008. A secondary peak in NDP rates was observed again in February and March 2009 with values close to 20 mmol O_2_ m^−2^ d^−1^.

### Statistical analysis of the vertical and seasonal variability of sediment inorganic nutrient fractions

**Figure 8:**
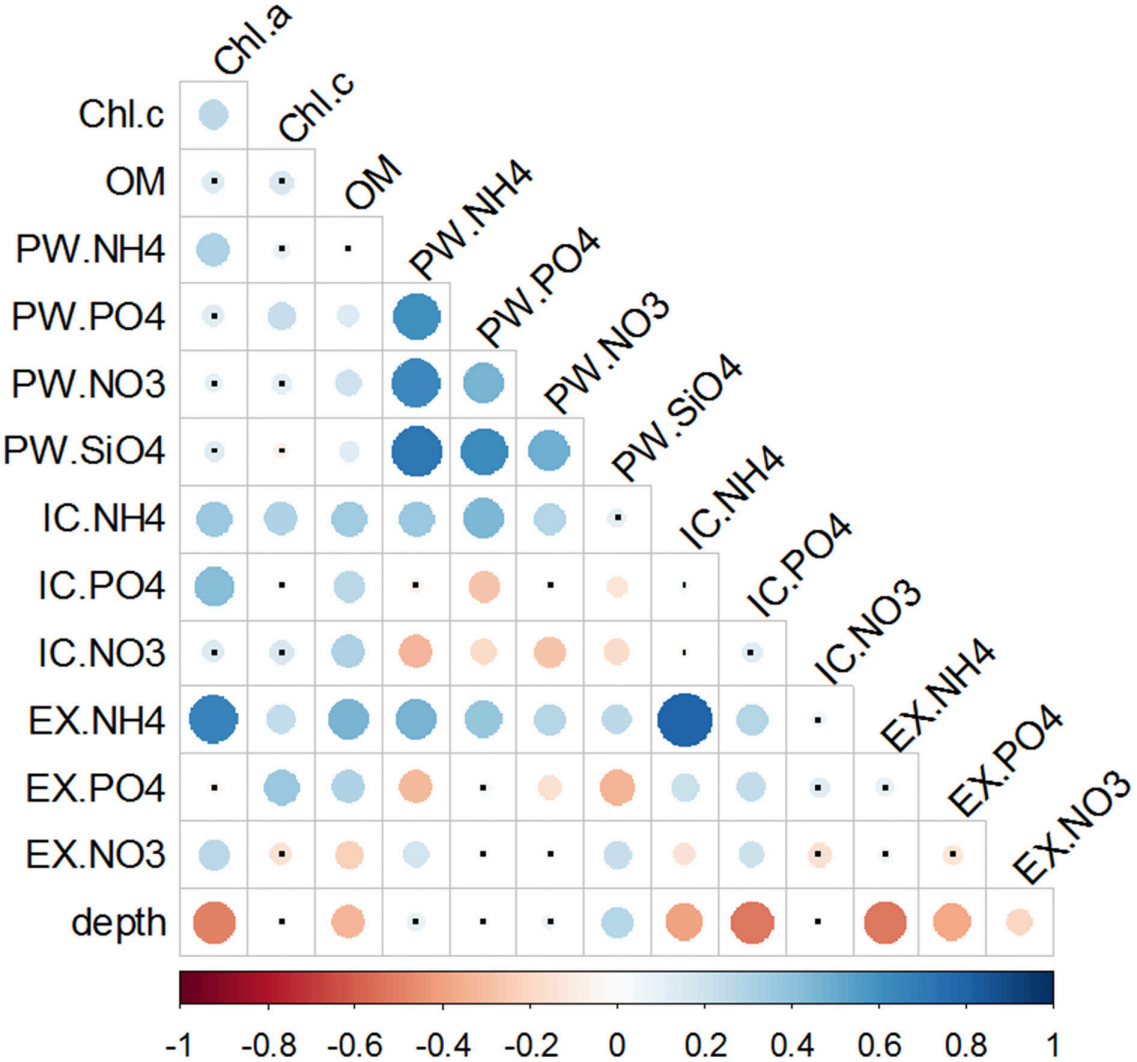
**Correlation matrix of chlorophylls and nutrient pools**. Data used for correlations were the full data set of triplicate monthly profiles. Color bar represents the Spearman correlation coefficient with blue indicating a positive and red a negative relationship. Size and tone of the dots is directly related to the magnitude of correlation. Non-significant correlations are labeled with a small black dot.

Nutrients were positively correlated between each other only within the PW fraction (Figure 8). When looking across the different pools, all three ammonium sediment fractions strongly correlated with each other, as well as with both Chlorophylls a and c, and OM. Phosphate fractions also significantly correlated with both Chlorophylls and OM. However, correlations between the different Phosphate fractions varied; ${\text{PW-PO}}_{4}^{3-}$ was inversely correlated with ${\text{IC-PO}}_{4}^{3-}$, whereas no correlation was found between ${\text{PW-PO}}_{4}^{3-}$ and ${\text{EX-PO}}_{4}^{3-}$. Nitrate fractions were both positively (${\text{PW-NO}}_{3}^{-}$ and ${\text{IC-NO}}_{3}^{-}$) and negatively (${\text{EX-NO}}_{3}^{-}$) correlated with OM content. Only ${\text{EX-NO}}_{3}^{-}$ fraction showed a significant correlation with Chl *a*. Within the nitrate pools, the only significant relation was found the negative correlation between ${\text{PW-NO}}_{3}^{-}$ and ${\text{IC-NO}}_{3}^{-}$.

**Figure 9:**
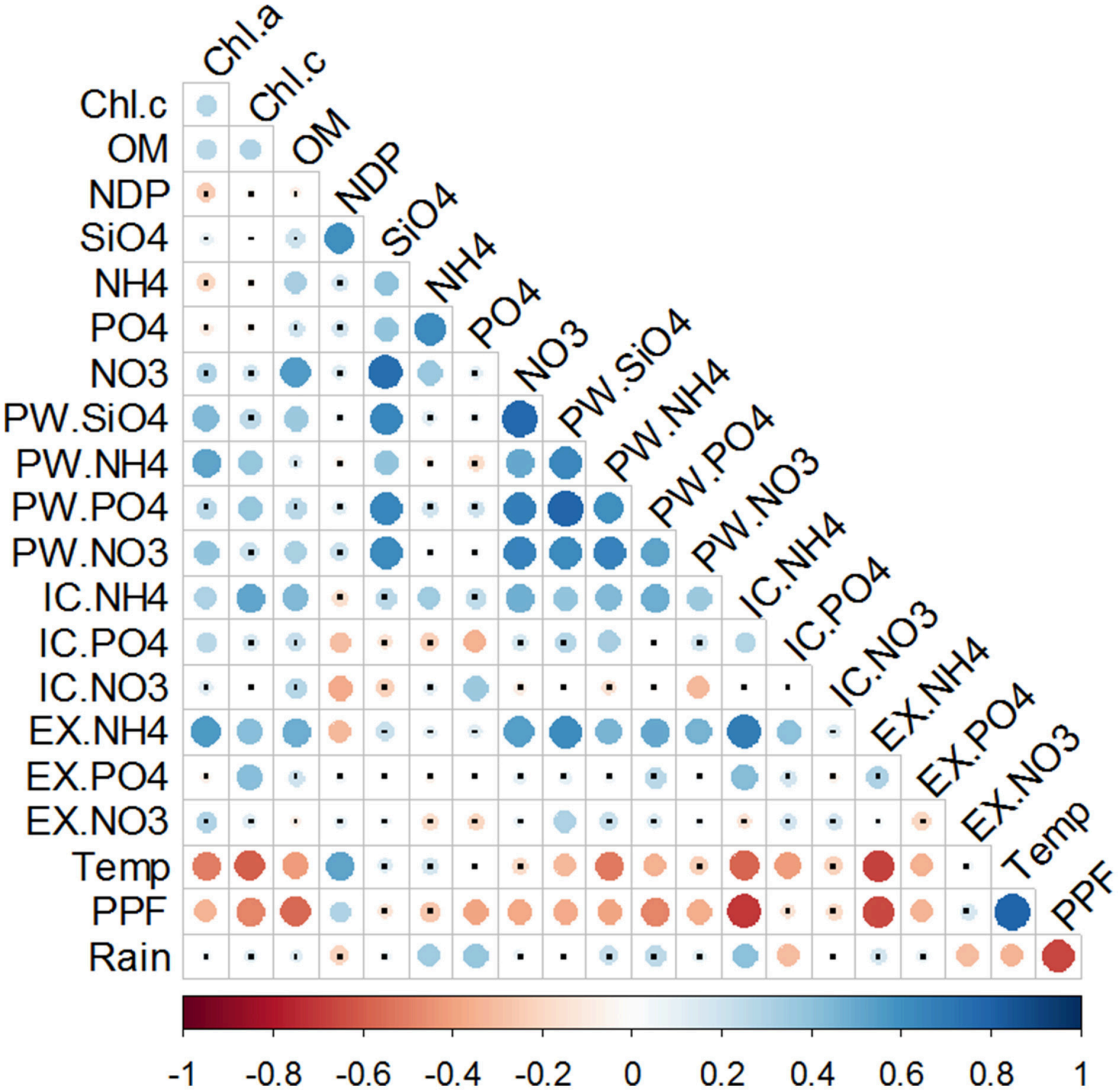
**Correlation matrix of the integrated variables in the first 4 mm of the sediment, water column nutrient concentrations, meteorological data and Net metabolism**. Chl.*a* and Chl.c represent the integrated pigment values in the first 4 mm of the sediment. ${\text{SiO}}_{4}^{4-}$, ${\text{NH}}_{4}^{+}$, ${\text{PO}}_{4}^{3-}$, and ${\text{NO}}_{3}^{-}$ represent the nutrient concentration in the water column. PW, IC, and EX stand for porewater, intracellular, and exchangeable pools, respectively. Temperature (Temp), photosynthetic photon flux (PPF), and Rain were obtained from a meteorological station localized in the outer part of the Bay. Potential Net Daily Production (NDP) was measured as the oxygen fluxes at the sediment-water interface. Color bar represents the Spearman correlation coefficient with blue data for positive and red for negative relationship. Size and tone of the dots is directly related to the magnitude of the correlation. Non-significant correlations are labeled with a small black dot.

In order to exclude the vertical variability, a second round of correlation analysis was performed between the sediment variables integrated in the top sediment layers (0–4 mm), where most of the changes were observed, and water column or rate variables (Figure 9). Silicate and phosphate in the water column were positively correlated with Chl *c* content in the surface sediment. Silicate was also the only nutrient that correlated with NDP. Water column silicate and nitrate correlated with ${\text{PW-SiO}}_{4}^{4-}$ and ${\text{PW-NO}}_{3}^{-}$, respectively, but no such relations could be observed for phosphate and ammonium. Rainfall showed a positive correlation with the nutrients with the lowest concentrations overall (phosphate and ammonium), but not with the most abundant ones (nitrate and silicate). Same as before, nutrients in the PW fraction correlated with each other and were all positively correlated with chlorophylls. Between fractions, all three ammonium pools showed positive correlations, but not phosphate and nitrate. An inverse correlation was found between ${\text{PW-NO}}_{3}^{-}$ and ${\text{IC-NO}}_{3}^{-}$.

Chlorophylls content was correlated with the IC and EX fractions of ammonium and phosphate, whereas no relation was found with nitrate fractions. NDP showed an inverse correlation with ${\text{IC-NO}}_{3}^{-}$ and ${\text{IC-PO}}_{4}^{3-}$ and a direct relationship with temperature and irradiance. All the other variables (namely nutrient fractions, pigments and organic matter content) were inversely correlated with air temperature and irradiance, with variable degrees of significance (Figure 9).

## Discussion

### Microphytobenthos seasonality and inorganic nutrients dynamics in the pore water and water column

Chl *a* concentration, an estimate of MPB abundance (Macintyre et al., 1996), can vary over an annual cycle as a function of abiotic factors such as temperature, light, and nutrients concentration, or disturbance events such as grazing and resuspension (Thornton et al., 2002). The relative effect of these factors produces the differences observed in sediment chlorophyll standing stocks between sampling sites. In the Bay of Cádiz, Chl *a* showed large variations at the sediment surface over the sampling period. Values ranged from 1.7 to 36 μg cm^−3^, being similar to those measured previously in the area (García-Robledo et al., 2010; Bohórquez et al., 2013) or in other temperate regions (Brotas and Plante-Cuny, 1998; Wolfstein et al., 2000; Du et al., 2009). The seasonal pattern of MPB abundance observed in the Bay of Cádiz, with minimum chlorophyll values in summer and highest in early winter is typical of similar latitude sediments (Brito et al., 2013), and is only occasionally found at higher latitude systems (Stief et al., 2013). In the latter, typically, an annual uni-modal maximum occurs in summer, which is usually attributed to the higher light availability and temperature during that period (de Jong and de Jonge, 1995; Wolfstein et al., 2000; Kang et al., 2006; Spilmont et al., 2006). In the Bay of Cádiz, however, pigment concentrations were negatively correlated with both irradiance and temperature (Figure 9). Thermo-inhibition and excessive sediment desiccation during emersion in summer (Blanchard et al., 1997; Morris and Kromkamp, 2003) and photoinhibition (Blanchard et al., 2004; Serôdio et al., 2008) probably affect MPB negatively. In addition, the higher grazer activity usually observed during spring and summer might also reduce MPB abundance during those periods (Gall and Blanchard, 1995; Pinckney et al., 2003).

In addition to the above, MPB abundance also depends on the nutrient supply from the water column. Higher Chl *a* concentrations are often found during winter months, when the runoff and leaching caused by rainfall is higher (Underwood and Paterson, 1993; Du et al., 2009; Stief et al., 2013). Water column nutrients in the Bay of Cádiz followed this seasonal pattern with higher values in autumn and winter (Figure 3), when precipitation is also highest in the area (Figure S1). Notwithstanding, despite of the high urbanization of the area, annual mean water column nutrient concentrations are low (present dataset; Morris et al., 2009; Papaspyrou et al., 2013), and could thus control MPB abundance (Welker et al., 2002; Stief et al., 2013). Although, no correlation was found between water column nutrient concentrations and annual pigments maxima (Figure 9), NDP was positively correlated with water column silicate, suggesting that this nutrient can be limiting for MPB in the Cadiz Bay. On the other hand, the mismatch observed in the temporal evolution of pigments and NDP, in particular the high NDP but low Chl *a* and Chl *c* values during the summer months, could be partially due to changes in the Chl *a* to carbon ratio, resulting in higher biomass with lower Chlorophyll (de Jonge, 1980) or a higher grazing activity during summer as mentioned above, which may limit the abundance of highly active MPB.

Porewater nutrients can be a significant nutrient source for MPB which in turn can largely control their availability in the porewater (Rysgaard et al., 1995; Risgaard-Petersen, 2003). As a result, positive, negative, or a lack of correlation have been found between Chl *a* and porewater nutrients (Du et al., 2009 and references therein). In Cadiz Bay, we found significant positive correlations between seasonal changes in Chl *a* and Chl *c* and PW nutrients, but not with water column nutrients (Figure 9). This fact supports the importance of pore water nutrients for MPB dynamics. Silicate in particular seems act as a limiting nutrient in the Bay of Cadiz; in addition to the aforementioned relation between NDP and water column silicate, sediment pigments content correlated with ${\text{PW-SiO}}_{4}^{4-}$. Given that the intertidal MPB community in the Bay of Cadiz is usually dominated by diatoms (García-Robledo et al., 2012), silicate seems to control to a large extent the MPB dynamics in the area due to its role for diatom growth.

### Intracellular nutrients in the intertidal sediments of Cadiz Bay

Intracellular nutrients in the intertidal sediments of the Cadiz Bay clearly accumulated during certain periods of time (Figures 1, 3). Seasonal variations of IC nutrients have been studied previously only in two coastal environments: in Cadiz Bay, where winter and summer IC nutrient concentrations correlated with MPB abundance (García-Robledo et al., 2010), and in the Wadden Sea, where the variation of the ${\text{IC-NO}}_{3}^{-}$ pool in intertidal sediments along 1 year was related to the water column nitrate concentration and the benthic diatoms abundance (Stief et al., 2013). In the present study, the highest accumulations of ${\text{IC-NH}}_{4}^{+}$ and ${\text{IC-PO}}_{4}^{3-}$ were found in the first few millimeters of the sediment during autumn 2008, when maximum Chl *a* and Chl *c* values were also measured, suggesting a direct relation between IC pools and MPB abundance. In contrast, the ${\text{IC-NO}}_{3}^{-}$ pool was more variable and no clear relationship with Chl *a*, such as the one described by Stief et al. (2013) for the Wadden Sea, was found here, suggesting a more complex regulation of this intracellular nutrient pool in the Bay of Cadiz.

The intracellular accumulation of any inorganic nutrient is the result of the balance between nutrient uptake, strongly dependent on the external supply, and its assimilation into organic compounds. In addition, the ${\text{IC-NO}}_{3}^{-}$ pool can be used by diatoms for dissimilatory nitrate reduction (Kamp et al., 2011), a major pathway for nitrate removal in some areas (Stief et al., 2013; Marchant et al., 2014). The ${\text{IC-NO}}_{3}^{-}$ content of the sediment in the Wadden Sea (0–20 nmol cm^−3^, with a peak of 65 nmol cm^−3^ during the diatom bloom) was similar to the one we measured in Cadiz Bay. However, the low nutrient concentrations in the water column and the succession of ${\text{IC-NO}}_{3}^{-}$, NDP, and Chl *a* peaks suggest that the intracellular nutrient pool was mainly used for assimilation and growth of MPB community rather than dissimilatory processes. Nevertheless, rates of dissimilatory reduction of nitrate to ammonium have not been quantified for our area. This process could be important for N cycling in Cadiz Bay sediments and should be taken into account in future investigations in the area.

In addition to diatoms, other microorganisms such as *Thioploca, Beggiatoa*, and foraminifera can accumulate millimolar concentrations of nitrate in intracellular vacuoles (Fossing et al., 1995; Sayama et al., 2005; Piña-Ochoa et al., 2010; Kamp et al., 2015). Although these microorganisms could contribute significantly to or even dominate the ${\text{IC-NO}}_{3}^{-}$ pool in some surface sediments, we believe that this is not the case in the inner Cadiz Bay. *Thioploca* and *Beggiatoa* are usually found in organic-rich sulfidic sediments but these conditions are not common in the iron-rich intertidal sediments of Cadiz Bay (Jiménez-Arias et al., 2016). Of the foraminifera groups found in the sediments of Cadiz Bay some are known to accumulate nitrate and perform denitrification; however, their abundance was very low and no relationship with water column or porewater nitrate was established (Papaspyrou et al., 2013). Therefore, the ${\text{IC-NO}}_{3}^{-}$ pool in the intertidal sediments of the inner Cadiz Bay is most likely related to benthic microalgae.

Both ${\text{IC-NH}}_{4}^{+}$ and ${\text{IC-PO}}_{4}^{3-}$ showed a positive correlation with Chl *a* and Chl *c*, both on the vertical and the temporal scale, suggesting that microbenthic community, in addition to nitrate, accumulate intracellularly ammonium and phosphate (Figures 8, 9). Accumulation of ${\text{IC-NH}}_{4}^{+}$ has been previously described for planktonic diatoms (Dortch, 1982; Thoresen et al., 1982); this accumulated ammonium is also detected in the sediment after the sedimentation of a phytoplanktonic bloom (Lomstein et al., 1990). In Cadiz Bay, high ${\text{IC-NH}}_{4}^{+}$ concentrations were measured with seasonal differences between summer and winter being clearly related to MPB abundance (García-Robledo et al., 2010). Similarly to ammonium, accumulation of phosphate has been shown in planktonic diatoms (Miyata et al., 1986), cyanobacteria (Mateo et al., 2006), and more recently in benthic diatoms (García-Robledo et al., 2010; Yamaguchi et al., 2015).

MPB might accumulate ammonium and phosphate to use them for assimilation and growth. In order to evaluate the relevance of these pools for the growth of MPB, the minimal algal nutrient demand was estimated using the Chl *a* increase during autumn 2008. Chl *a* increased from 1.4 μg cm^−2^ in August 2008 to 15.9 μg cm^−2^ in December 2008. Considering N:Chl *a* and P:Chl *a* ratios of 5.58 and 1.15, respectively -measured for the benthic diatom *Navicula* sp. (Montani et al., 2003)—a minimum of 80.9 nmol N cm^−2^ and 16.7 nmol P cm^−2^ would be required to produce the measured Chl *a* peak. Similarly, the Chl *a* peak measured in March 2009, a net increase of 7.3 μg Chl *a* cm^−2^ from January, corresponded to a demand of 40.9 nmol N cm^−2^ and 8.4 nmol P cm^−2^. Those values are likely minimum estimations as no losses due to grazing, resuspension or mortality are considered.

For phosphorus, the requirement estimates obtained were quite similar to the 13 nmol ${\text{IC-PO}}_{4}^{3-}$ cm^−2^ measured in November 2008, just before the Chl *a* peak in December 2008, and the 7 nmol ${\text{IC-PO}}_{4}^{3-}$ cm^−2^ measured in February 2009, just before the peak in March 2009. Thus, it seems plausible that MPB accumulated phosphate intracellularly to be used for net growth, independently of the external nutrient supply. Although our calculations on phosphorus demand agree well with the ${\text{IC-PO}}_{4}^{3-}$ pool, MPB can also accumulate large quantities of phosphorus as polyphosphate which could have been broken down to some extent by the extraction procedure and measured as phosphate. In addition, other organisms such as bacteria could have contributed to the phosphate (Aller, 1994) or polyphosphates measured in the ${\text{IC-PO}}_{4}^{3-}$ pool, the latter being a relevant phosphorus pool in freshwater sediments (Gächter et al., 1988).

Contrary to phosphorus, the ${\text{IC-NH}}_{4}^{+}$ pool exceeded by far the estimated requirements for growth, being as high as 1400 nmol cm^−2^ during November 2008. Therefore, despite the significant correlation between IC-NH_4_+ and photosynthetic pigments (Figures 8, 9), probably other ammonium pools, additional to the MPB intracellular one, were contributing to the large ${\text{IC-NH}}_{4}^{+}$ measured in the sediments of Cadiz Bay. Bacterial abundance is generally well coupled to Chl *a* in sediments (Middelburg et al., 2000), therefore it is reasonable to attribute to bacteria a large fraction of the ${\text{IC-NH}}_{4}^{+}$ pool. Aller (1994) found concentrations of lysable ammonium as high as 7 μmol ${\text{NH}}_{4}^{+}$ g^−1^ in aphotic sediments and a direct correlation with bacterial abundance. Considering a sediment density of 2.5 g cm^−3^, this ${\text{IC-NH}}_{4}^{+}$ content corresponds to 2800 nmol cm^−3^. The magnitude of this content is similar to the one measured in the present study during the Chl *a* peak ocurring in December 2008. In addition, other sources of nutrients, such as micro- and meiofauna, as well as different types of phytodetritus, could co-vary with MPB and may also have contributed to the ${\text{IC-NH}}_{4}^{+}$ pool (Lomstein et al., 1990; Corzo et al., 2009). In the future, an effort should be made to distinguish the sources contributing to the measured IC pools, if we wish to understand the observed differences in the temporal and vertical distribution of the intracellular pools among sites and establish valid links between IC pools and MPB primary production and biomass.

### Exchangeable nutrients and the relative importance of each nutrient pool

Exchangeable ammonium and phosphate are considered important nutrient reservoirs in marine sediments (Lomstein et al., 1990; Laima, 1992; Aller, 1994; Yamaguchi et al., 2015). Accordingly, in Cadiz Bay, ${\text{EX-NH}}_{4}^{+}$ and ${\text{EX-PO}}_{4}^{3-}$ content was on average 15 and 10 times higher, respectively, than the corresponding porewater one, despite the ${\text{EX-NH}}_{4}^{+}$ and ${\text{-PO}}_{4}^{3-}$ variations throughout the year and with depth in the sediment (Figures 3, 4). Concentrations were generally higher in the upper part of the sediment, decreasing with depth, and higher during late autumn months, when chlorophylls and OM were also highest. EX nutrients concentration measured in Cadiz Bay sediments are similar to those in other muddy sediments from coastal areas, where a large temporal and spatial variability was also reported (Laima, 1992; Van Raaphorst and Malschaert, 1996). For example, Laima (1992) found higher ${\text{EX-NH}}_{4}^{+}$ following the phytoplankton sedimentation in Aarhus Bay (Denmark) with values up to 1400 nmol cm^−3^ at the sediment surface, similar to the ones measured here. In the Mondego estuary (Portugal), integrated ${\text{EX-PO}}_{4}^{3-}$ values varied from 40 to 1380 mmol m^−2^ (Coelho et al., 2004), corresponding to 0.1–2.8 nmol cm^−2^ (assuming homogeneous distribution and rescaling to 4 mm of sediment), similar to the integrated values measured here. Overall, the ${\text{EX-PO}}_{4}^{3-}$ in Cadiz Bay followed the same evolution than the ${\text{EX-NH}}_{4}^{+}$ and suggests a common regulation.

Among other variables, loosely adsorbed ions concentration depends on porewater concentration, salinity, availability of binding sites, and temperature (Laima, 1992; Van Raaphorst and Malschaert, 1996; Coelho et al., 2004). Neither changes in granulometry, and as consequence in the amount of clay particles in the sediment (and available sorption sites), nor in salinity occur in Cadiz Bay during the course of a year, since no rivers flow into the bay. Organic matter, on the other hand, showed a clear seasonal pattern and was positively correlated with both ${\text{EX-NH}}_{4}^{+}$ and ${\text{EX-PO}}_{4}^{3-}$ (Figures 8, 9). Sediment organic matter can control exchangeable pools in several ways. First, OM provides additional sorption sites for cations and can be the main source of binding sites for ammonium (Van Raaphorst and Malschaert, 1996). Second, OM acts as a source of nutrients during the remineralization process. Finally, organic ions can compete with inorganic anions for Fe and Al oxides adsorption sites, in the same way as it has been described for soils (Johnson and Todd, 1983; Hue, 1991; Gu et al., 1995).

Binding of nitrate to sediments is theoretically possible (Wang et al., 1987) and has been described both in soils (Katou et al., 1996; Matson et al., 1999) and in marine sediments already a few decades ago (Garber, 1984); however, the exchangeable nitrate fraction has not been investigated in detail. In marine environments, competition with Cl^−^ and other anions reduces the availability of free sorption sites, leading to the misconception than an absorbed nitrate pool cannot exist. However, in the few cases where the ${\text{EX-NO}}_{3}^{-}$ pool has been quantified, it has been found to be significant (Garber, 1984; Lomstein et al., 1990; Papaspyrou et al., 2014). Lomstein et al. (1990) found high ${\text{EX-NO}}_{3}^{-}$ concentrations at the sediment surface of Aarhus Bay (up to 100 nmol cm^−3^) and a quick decrease with depth. In the sediments of the hyper-nutrified Colne estuary (UK), high ${\text{EX-NO}}_{3}^{-}$ content was found several centimeters down in the sediment, with values usually above 10 nmol g ww^−1^; although, no distinction was made between IC and EX pools (Papaspyrou et al., 2014). In both studies, the ${\text{EX-NO}}_{3}^{-}$ pool at the sediment surface was shown to be several times higher than ${\text{PW-NO}}_{3}^{-}$. In like manner, the ${\text{EX-NO}}_{3}^{-}$ pool in Cadiz Bay was on average seven times higher than the porewater one, being up to 50 times higher at times. In fact, this ${\text{EX-NO}}_{3}^{-}$ pool was the main nitrate pool in Cadiz Bay sediments. The consolidated nature of the muddy sediment in our area suggests that the source is probably not nitrate introduced from the water column by advection, given also the generally low water column nitrate concentration. For the surface sediment, where oxygen penetrates (Papaspyrou et al., 2013), a potential source of nitrate could be bacterial nitrification, whereas for deeper layers, coupled nitrification-denitrification stimulated by the bioirrigation activities of micro- and meiofauna could be also possible (Bonaglia et al., 2014). However, the source of the ${\text{EX-NO}}_{3}^{-}$ at depth remains to be determined.

The ${\text{EX-NO}}_{3}^{-}$ fraction showed a larger variability with several peaks throughout the sampling period, compared to ${\text{EX-NH}}_{4}^{+}$ and ${\text{EX-NH}}_{4}^{3-}$. In addition, contrary to the other two nutrients, ${\text{EX-NO}}_{3}^{-}$ was inversely correlated to the vertical distribution of OM content in the sediment and seemed to be more abundant after the Chl maximum values. These facts suggest that that ${\text{EX-NO}}_{3}^{-}$ is more dynamic than ${\text{EX-NH}}_{4}^{+}$ and ${\text{EX-PO}}_{4}^{3-}$ with additional factors controlling this pool. Although, an increase in organic matter following the Chl *a* peak would provide more sorption sites for ammonium, the contrary would be true for nitrate due to the competition with organic ions (Schlesinger and Bernhardt, 2013). In addition, the high nitrogen demand by the MPB community could also result in a decrease of the ${\text{EX-NO}}_{3}^{-}$ pool. Regardless of the mechanisms in action, the existence and variability of the ${\text{EX-NO}}_{3}^{-}$ pool is not only relevant for the dynamics of the MPB. As suggested by Papaspyrou et al. (2014), the ${\text{EX-NO}}_{3}^{-}$ pool could be used by other components of the microbial community and may be supporting dissimilatory nitrate reduction processes at sediment depths where ${\text{PW-NO}}_{3}^{-}$ has been fully consumed. In the Bay of Cadiz, this pool seemed to be the most abundant and peaked when MPB abundance decreased. It is evident that further investigations about the dynamics of ${\text{EX-NO}}_{3}^{-}$ and its role for nitrogen cycling are needed. The high concentrations usually found compared with the PW one and the apparent fast response to environmental factors suggest that this fraction is an important nitrate pool in marine sediments that potentially should be considered in biogeochemical and microbial ecology studies of sediment nitrogen cycling.

## Conclusions

The simultaneous study of the three nutrient pools, PW, IC, and EX, in the upper cm of an intertidal sediment in Cadiz Bay over the course of a year showed that all three pools are highly dynamic and that their content is correlated with the distribution of microphytobenthos (MPB). Although, no data exist at present on the internal cycling of the different pools, our data suggest that IC and EX pools represent the major fraction of sediment total nutrient pools and that these could control to a large extent the dynamics of MPB in intertidal sediments. Refining various methodological aspects in order to define better the origins of each pool is necessary (e.g., distinguishing between microalgae and bacteria contributions), in order to understand the links not only between IC and EX pools and MPB primary production and biomass, but also the degree of exchange between the pools themselves. Finally, one of the most abundant pools observed here, that of ${\text{EX-NO}}_{3}^{-}$, which is usually not considered, should be taken into account in future studies of sediment nitrogen cycling given its potential importance for various nitrogen processes. It is clear that we urgently need to expand our database from areas with different sediment characteristics and MPB cycle patterns to shed some more light on the sediment “black box”; most likely we will have to revise and redefine several aspects of benthic nutrient dynamics.

## Author contributions

All the authors designed the study and carried out the samplings. EG and SP analyzed the samples and the data. All authors interpreted the data and wrote the manuscript.

## Funding

The study was funded by projects, CTM2013-43857-R from the Spanish Ministry of Economy and Competitiveness and P11-RNM-7199 from Consejería de Innovación, Ciencia y Empresa, Junta de Andalucía. JB was funded by a fellowship grant from the Ministry of Education and Science (BES- 2010-035711) and JJ by a University of Cádiz Plan Propio fellowship grant (2010-063). SP was funded by a JAE-Doc fellowship (Programa JAE, JAE-Doc109, Spanish National Research Council) and a Marie Curie ERG action (NITRICOS, 235005, European Union).

### Conflict of interest statement

The authors declare that the research was conducted in the absence of any commercial or financial relationships that could be construed as a potential conflict of interest.
