# Supplementary material for: Dynamics of Inorganic Nutrients in Intertidal Sediments: Porewater, Exchangeable, and Intracellular Pools
Source: Front Microbiol. 2016 May 26;7:761. doi: 10.3389/fmicb.2016.00761 (PMC4880585; doi:10.3389/fmicb.2016.00761)
Supplement: Supplementary file 1 [file DataSheet1.docx]

# Supplementary Material.

Figure S 1. Meteorological data from El Puerto de Santa Maria Meteorological Station (El Puerto de Santa Maria) localized in the outer part of Cadiz Bay. Data are mean of 15 days before the sampling for temperature and Photosynthetic Photon Flux (PPF) whereas the Rainfall represents the accumulated rain between sampling dates, expressed in mm. Photosynthetic Photon Flux (PPF) was calculated as daily PPF dose divided by the daytime length to be expressed in µmol photons m^-2^ s^-1^.
